# Vertical Canopy Profile and the Impact of Branches on Soybean Seed Composition

**DOI:** 10.3389/fpls.2021.725767

**Published:** 2021-09-08

**Authors:** Luiz Henrique Moro Rosso, André Froes de Borja Reis, Ignacio Antonio Ciampitti

**Affiliations:** Department of Agronomy, Kansas State University, Manhattan, KS, United States

**Keywords:** protein, oil, amino acids, fatty acids, seed yield

## Abstract

Soybean [*Glycine max* (L.) Merr.] seeds are of global importance for human and animal nutrition due to their high protein and oil concentrations, and their complete amino acid (AA) and fatty acid (FA) profiles. However, a detailed description of seed composition at different canopy portions (i.e., main stem and branch nodes) is currently lacking in scientific literature. This study aims to (1) characterize seed yield and composition (protein, oil, AA, and FA) at the main stem (exploring a vertical canopy profile) and stem branches and (2) quantify the impact of canopy yield allocation on seed composition, focusing on branches as a potential contributor for higher yields. Four genotypes were field-grown during the 2018 and 2019 seasons, with seeds manually harvested from all the branches and three main stem segments (lower, middle, and upper). Seed samples were analyzed for seed yield (Mg/ha), seed size (mg/seed), protein and oil content (mg/seed) and their respective concentrations (g/kg), and AA and FA concentrations within protein and oil (g/100 g), herein called abundance. The upper main stem produced greater protein (25%) and oil (15%) content relative to the lower section; however, oil concentration increased from top to bottom while protein concentration followed opposite vertical gradient. Limiting AAs (lysine, cysteine, methionine, threonine, and tryptophan) were more abundant in the lower main stem, while the oleic/(linoleic + linolenic) ratio was greater in the upper segment. Overall, branches produced seeds with inferior nutritional quality than the main stem. However, the contribution of branches to yield (%) was positively related to limiting AA abundance and oil concentration across soybean genotypes. Future research studies should consider the morphological process of stem branching as a critical factor intimately involved with soybean seed composition across environments, genotypes, and management practices.

## Introduction

High concentrations of seed protein and oil have expanded soybean [*Glycine max* (L.) Merr.] production worldwide. In 2018, 345 Tg of soybean seeds were produced (FAO, 2021). Considering a safe protein intake of ~60 g/adult/day (WHO/FAO/UNU, 2007), soybeans alone can supply roughly 75% of the global protein need and contribute to 30% of global vegetable oil production (FAO, 2021). In the United States (US), dry basis protein and oil concentration are about 400 and 215 g/kg, respectively (Rotundo et al., 2016). Environmental conditions are known to modify protein and oil concentrations by roughly 20% (Rotundo and Westgate, 2009), with these factors dominating the variation on soybean seed composition (Assefa et al., 2019). However, changes in seed composition within the plant canopy (Collins and Cartter, 1956) have received less attention, especially considering seeds from the branches.

Modern soybean genotypes not only produce high yields under high plant density but also compensate for the absence of plants with enhanced branching (Suhre et al., 2014). This flexibility might favor yield stability (Agudamu and Shiraiwa, 2016), especially under adverse conditions of stand establishment in northern latitudes (Lamichhane et al., 2020). Along with yield increase, soybeans have been reported to have decreased protein and increased oil concentration (Rincker et al., 2014). However, it is unclear that how branches contributed to the yield-protein-oil relationship in soybeans. Furthermore, the concentration of a seed component is a consequence of its content, which depends on assimilate supply (Rotundo et al., 2009) and varies within the canopy. Despite genotype leaf characteristics, greater net radiation is found in the upper section of the canopy, and greater temperature, smaller vapor-pressure deficit (VPD), and smaller carbon dioxide (CO_2_) concentration, all relative to lower canopy (Baldocchi et al., 1983, 1985).

Genetics and the environment have a great influence on stem branching in soybeans (Shim et al., 2017, 2019). Remarkably, low plant density enhances branching (Carpenter and Board, 1997), possibly associated with radiation quality (e.g., red to far-red ratio) at the ground level (Toyota et al., 2017). Branch leaves unroll about 30 days after sowing and develop under continuous shading, presenting thinner leaves compared to the main stem (Koller, 1972). In addition, branch nodes have late flowering and pod set but similar physiological maturity compared to the main stem (Munier-Jolain et al., 1994). This internal ontogenesis variation relates to reproductive abortion, seed-filling rate and duration, and yield (Egli and Bruening, 2006a,b). During the seed filling, 30% of a leaf carbon (C) assimilate is remobilized to pods on the same node and the other 30–40% to the four neighbor nodes (Stephenson and Wilson, 1977). Therefore, differences in source for C assimilation might prevail on the seed composition of a stem segment, mainly for protein due to the strong nitrogen (N) remobilization process (Sinclair and De Wit, 1975).

At physiological maturity, upper main stem nodes have greater protein and lesser oil concentration than the lower main stem (Sharma et al., 2013), but little is known about the concentration of seed components in the branches. Under low plant densities, this vertical gradient for seed composition is reduced but still maintained (Huber et al., 2016). Protein and oil vertical profiles are consistent regardless of soybean growth type (determinate or indeterminate) and genotype protein level (Escalante and Wilcox, 1993a,b). A few reports have explored the concentration of amino acids (AAs) and fatty acids (FAs) as a measure of the soybean nutritional value. Bennett et al. (2003) found a greater concentration of sulfur-containing AAs in the lower main stem seeds, while seeds in the upper main stem nodes presented higher oleic acid. Greater oleic concentration in the top main stem nodes was confirmed by Bellaloui and Gillen (2010), contributing to heat stability and shelf life for food preparation and biodiesel industry (Carrera and Dardanelli, 2017). Sulfur AAs (cysteine and methionine) are among the five limiting AAs (with lysine, threonine, and tryptophan) often supplemented in monogastric dietary (Thakur and Hurburgh, 2007).

Although a majority of the soybean industry does not reward superior nutritional quality (protein, oil, AA, and FA) (Brumm and Hurburgh, 2006), the increasing demand for sustainable food production could disseminate premium payments, promote seed-quality segregation at the field level, and enhance competitiveness, and marketability. Differences in seed yield and composition from the main stem and branches should be explored to understand potential unintended changes in these critical plant traits at the whole plant level. Following this rationale, the aims of this study were to (1) characterize the seed yield and composition (protein, oil, AA, and FA) at three segments of the main stem (vertical canopy profile) and stem branches; and (2) quantify the impact of canopy yield allocation on seed composition, focusing on branches as a potential contributor for high yields in modern genotypes.

## Materials and Methods

### Experimental Design and Growing Conditions

Field experiments were performed during the 2018 and 2019 growing seasons, at the Ashland Bottoms Agronomy Farm (39.14° North, 96.64° West, 315 m elevation, in Manhattan, Kansas, US) and Kansas River Valley Experimental Field (39.12° North, 95.92° West, 280 m elevation, in Rossville, Kansas, US), respectively. Conventional tillage was performed before sowing, and composite soil samples were collected to characterize texture and initial chemical properties (Table 1). Both fields have been under soybean-corn (*Zea mays*) rotation, and irrigation was not adopted during the growing season. Climate is classified as Cfa (humid subtropical) with evenly distributed precipitation throughout the year (Köppen, 2011).

**Table 1:** Soil and weather variables characterizing Ashland Bottoms 2018 and Rossville 2019 experimental sites.

**Variable**	**Ashland 2018**	**Rossville 2019**
Soil variables		
Soil texture, g kg^−1^		
Clay	180	173
Sand	280	300
Silt	540	527
Water pH	7.6	7.0
SOM, g kg^−1*a*^	21.0	15.0
NO_3_, mg dm^−3^	4.0	2.7
SO_4_, mg dm^−3^	1.0	2.3
P, mg dm^−3*b*^	90.2	43.0
Weather variables		
Mean temperature, °C	25.7	24.3
Maximum temperature, °C	32.2	29.7
Minimum temperature, °C	19.1	18.9
Solar radiation, MJ m^−2^ day^−1^	1687	1296
Evapotranspiration, mm^c^	754	474
Rainfall precipitation, mm	338	518
Precipitation SDI^d^	0.59	0.65
Relative humidity, %	59.7	77.5
Mean VPD, kPa^e^	1.65	0.73

*Composite soil samples were collected before sowing from a 0.15-m-depth layer, except for nitrate (NO_3_) and sulfate (SO_4_), measured from a 0.60-m-depth layer. Weather variables were summarized within the entire soybean growing season at each location. ^a^Soil organic matter via loss on ignition (LOI). ^b^Phosphorus extracted by Mehlich-3. ^c^Reference evapotranspiration. ^d^Shannon-diversity index (Bronikowski and Webb, 1996). ^e^Vapor-pressure deficit*.

Four genotypes with contrasting branching potential were selected: P31T11R [maturity group (MG) 3.1, released in 2014]; P34T43R2 (3.4, 2014); P35T58R (3.5, 2013); and P39T67R (3.9, 2013) (Corteva Agriscience, Johnston, Iowa, US). Experimental design followed a randomized complete block with four repetitions in both site-years. Treatment factors were (1) genotype (four levels) and (2) canopy portion from which seeds were produced (lower, middle, and upper main stem segments and the branches altogether). Herein, we use the term branches to represent a morphological structure accounting for a fraction of the seed yield, not the process of branching itself. Experimental plots consisted of six rows spaced 0.75 m and a plot size of 60 m^2^. The sowing dates were April 29, 2018, and June 9, 2019. All genotypes were sown at 300,000 seeds/ha that resulted in approximately 240,000 plants/ha at harvest time. Soybean seeds were inoculated before sowing with Vault HP Rhizobia Inoculant (BASF, Ludwigshafen, Germany) containing at least 3.0 × 10^9^ colony-forming unit/ml of *Bradyrhizobium japonicum*. Weeds, insects, and diseases were managed according to the best agronomic practices.

Weather variables were retrieved from the DAYMET database (Thornton et al., 2020) and summarized from soybean emergence (VE) to physiological maturity (R7 stage, one pod in the main stem had reached mature pod color) (Fehr and Caviness, 1977) according to Correndo et al. (2021) (Table 1). The soybean cycle reached ~120 days in 2018 and ~105 days in 2019. Differences in MG across the tested genotypes introduced an overall season-length variation of less than a week. The sowing date affected the seasonal weather conditions, with Rossville 2019 (late sowing) presenting lower temperatures and solar radiation. Ashland 2018 was less humid and had greater VPD and lower precipitation compared to Rossville 2019.

### Measurements and Laboratory Analysis

At harvest time (R8 stage), three central adjacent rows covering ~3.4 m^2^ were manually harvested from each plot. Main stems were divided into three segments (lower, middle, and upper), with five to six nodes in each segment. Cotyledonary and unifoliolate nodes were not considered due to the absence of pods. Branches were collected as a unique segment, adding up to the four canopy portions evaluated. Samples were machine threshed and taken to the laboratory for determining seed yield (Mg/ha) and seed weight (mg/seed, herein called seed size), both adjusted to 130 g/kg moisture basis. Seed number (1,000 seeds/m^2^) was calculated based on yield and seed size. Finally, seed samples (~500 g) were oven-dried (65°C) until constant weight is obtained and ground to 0.1 mm particle size. Protein, oil, AA, and FA concentrations were determined *via* near-infrared spectroscopy (NIR) using the Perten DA7200 Feed Analyzer (Perten Instruments, Stockholm, Sweden). The ground material was scanned between 1,000 and 2,500 nm wavelength, and normalized reflectance readings were used to estimate each seed component. The calibration method was based on Honigs et al. (1985) and evaluated with cross-validation using the coefficient of determination (*r*^2^). After accounting for protein and oil, the remaining seed size was classified as residue fraction, mostly carbohydrates.

Besides dry basis concentration (g/kg), seed components were expressed in content (mg/seed), a consequence of the seed-filling process, speaking to industry and agronomists. The AA and FA concentrations were expressed within protein (g/100 g protein) and oil (g/100 g oil), respectively, as a measure of abundance within each component (Gerde and White, 2008). Because NIR does not differentiate asparagine and aspartate, or glutamine and glutamate, these AAs were expressed as aspartic and glutamic acid, respectively. Therefore, the 20 primary AAs were analyzed as a total of 18 types and then added within three groups: (1) non-essential (alanine, arginine, aspartic acid, glutamic acid, glycine, proline, serine, and tyrosine); (2) essential non-limiting (isoleucine, leucine, histidine, phenylalanine, and valine); and (3) essential limiting (lysine, cysteine, methionine, threonine, and tryptophan) following Pfarr et al. (2018). The abundance of those five limiting AAs (LAAs, g/100 g protein) was considered the main descriptor of protein quality hereafter. Five FAs were determined: linoleic, oleic, palmitic, linolenic, and stearic. However, the oleic/(linoleic + linolenic) ratio was the main descriptor of oil quality (Gao et al., 2009). Whole plant and main stem data were calculated as the weighted average of the containing portions, considering respective yields.

### Statistical Analysis

Linear mixed models related dependent (e.g., yield and protein content) with independent variables (i.e., genotype and canopy portion). Those variables were tested using three models, considering different levels of the plant canopy: (1) whole plant (only testing the effect of genotype); (2) two canopy portions (also comparing main stem and branches); and (3) four canopy portions (lower, middle, and upper main stem segments and the branches). For the first model, genotype was the only fixed effect (four levels), with a random intercept for site-year, block, and block within site-year. For the other two models, fixed effects were canopy portion, genotype, and their interaction, with random effects also including genotype nested in block × site-year, because canopy portions were observed on the same plant sample. To investigate the relationship between seed yield of the whole plant or entire main stem and the contribution of branches to yield, a regression model was proposed across all soybean genotypes. In this case, seed yield (Mg/ha) was the dependent, and branch-yield contribution (%) was the independent variable, both continuous and with a random intercept for site-year.

Finally, and related to the second objective, seed yield of the whole plant and branch-yield contribution were tested as fixed effects (independent variables) describing protein and oil concentrations, LAA abundance, and oleic/(linoleic + linolenic) ratio (four dependent variables). A random intercept for site-year was also included, and dependent variables were considered at the whole plant level or by canopy portion (checking for interactions). This model intends to dissect the effect of branches from the effect of yield variation on soybean seed composition. The independent variables were centered (subtracted by the mean) and scaled (divided by the standard deviation) before model fitting and ANOVA. The center-scale transformation was performed due to contrasting variable magnitude, which could impair the hypothesis testing.

Statistical analysis was performed in the R software (R Core Team, 2020). For all variables, normality and homogeneity of variance were checked using Shapiro–Wilk and Bartlett's test, and data transformations were not employed. The *lme4* package (Bates et al., 2015) was used to fit the models, and the *car* package (Fox and Weisberg, 2019) was used to perform type III ANOVA. Significant effects represented *p* value < 0.05 (*F*-test). A protected Fisher's least significant difference (LSD) test was adopted for means comparison using the *multcomp* package (Hothorn et al., 2008). The least-square means (LSMEANS) were computed for all the treatment combinations. The figures presented in this manuscript were generated using the *ggplot2* package (Wickham, 2016).

## Results

### Seed Yield and Macrocomponent Content

**Figure 1:**
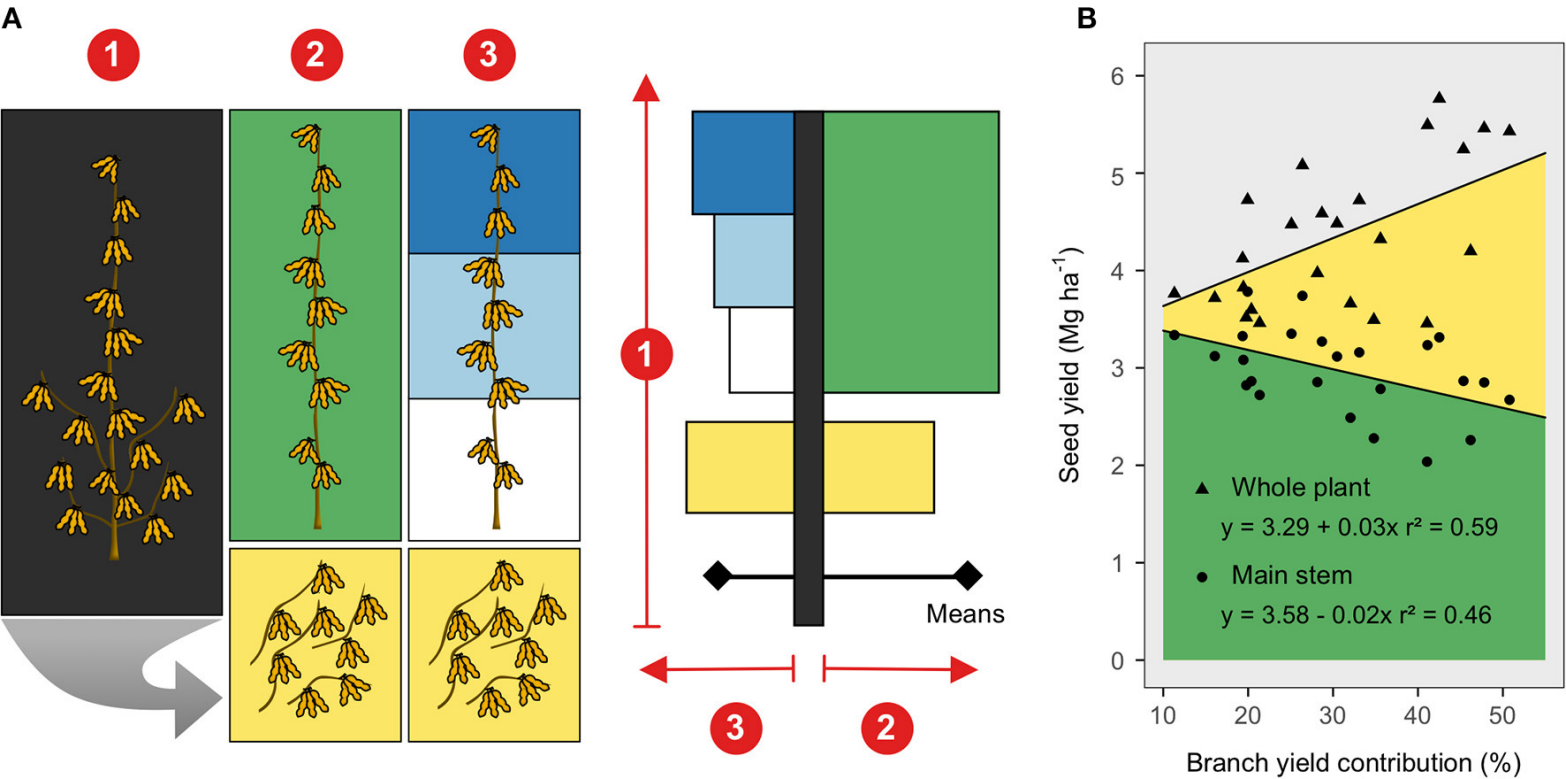
**(A)** Soybean seed harvesting and bar chart representation of observed variables at three canopy levels: (1) whole plant, (2) entire main stem (green color) and stem branches (yellow color), and (3) upper (dark blue), middle (light blue), lower (white color) main stem segments and the branches. The bar chart representation was meant to resemble a soybean plant, concisely depicting the linear mixed models testing genotype and canopy portion. **(B)** Seed yield from the main stem (green) and branches (yellow color) relative to the branch-yield contribution (%). Regression lines were fit across genotypes and considered a random intercept for site-year.

**Figure 2:**
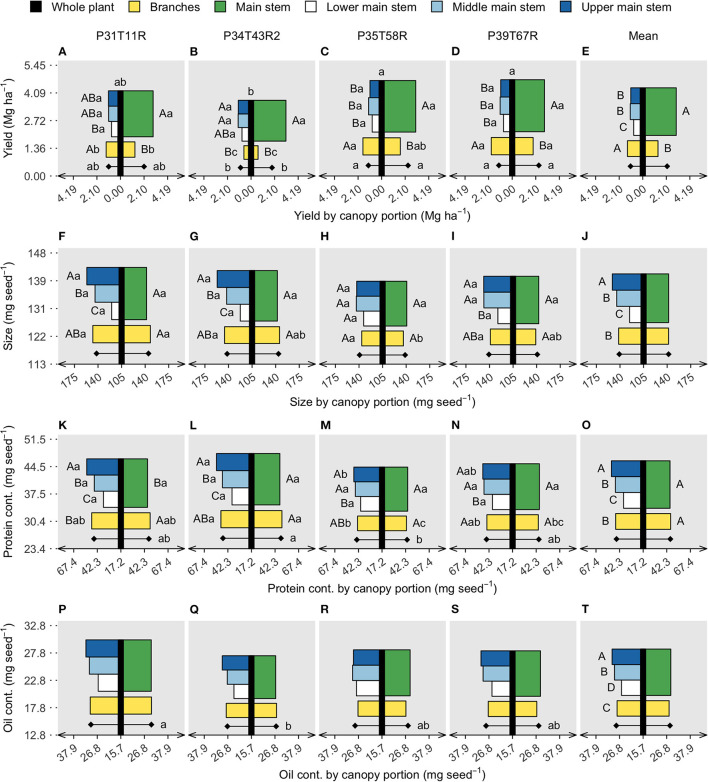
Soybean seed protein **(A–E)** and oil concentration **(F–J)**, limiting amino acids (LAAs) abundance **(K–O)**, and oleic/(linoleic + linolenic) ratio **(P–T)**. Vertical black bars refer to the whole plant data in the y axis, with lowercase letters on top comparing genotypes (model 1). Horizontal bars are centered on the black bar (x axis), referring to two canopy portions on the right side (main stem and branches) (model 2) and four canopy portions on the left side (lower, middle, and upper main stem segments and branches) (model 3). Diamonds represent the genotype mean of all canopy portions for models 2 and 3. Uppercase letters compare stem segments within genotype (interaction) or on the overall mean (portion effect). Lowercase letters compare genotypes within canopy portions (interaction) or on the genotype mean, diamonds (genotype effect). Each panel row portraits three linear models of a response variable, for the whole plant, two and four canopy portions. Absence of letters represents no significant difference (*p* < 0.05).

Results from the three linear mixed models, comparing genotypes and canopy portions, are displayed using bar charts that resemble a soybean plant (Figure 1A). Remarkably, across the tested soybean genotypes greater yields were attainable as the branch contribution to seed yield increased (Figure 1B), at the expense of seed yield derived from the main stem. At the whole plant level, seed yield differed among genotypes, ranging from 3.7 to 4.7 Mg/ha (Figures 2A–D), mainly driven by seed number rather than seed size (Supplementary Figures 1A–D). Although the main stem produced most of the yield (~70%), significant yield differences among genotypes were only captured in the branches, ranging from 0.6 to 1.9 Mg/ha. The high-yielding genotypes (P35T58R and P39T67R) produced greater seed yield coming from the branches (Figures 2C,D). Seed size in the main stem was also similar across genotypes but was variable in the branches (Figures 2F–I). The lower main stem yielded overall 25% less than the middle and upper (Figure 2E) segments, while branches yielded more than the main stem segments in the P35T58R and P39T67R (genotypes with high branch yield, ~37% contribution to yield), and the same or less in the P31T11R and P34T43R2 (genotypes with low branch yield, ~24% contribution to yield). Yield variations within the main stem were not connected to seed number, but to seed size, decreasing ~17% from top to bottom nodes (Figure 2J). On the other hand, the yield from branches was more proportional to changes in seed number, as seed size was similar to the entire main stem. The ANOVA coefficients for the mixed models testing genotype and canopy portion are shown in Supplementary Table 1.

Seeds from the upper main stem accumulated 25% more protein (Figure 2O) and 15% more oil (Figure 2T) contents than the lower main stem. However, the vertical gradient of protein content was attenuated for the soybean genotypes presenting high branch yield. Across the tested genotypes, protein content within the branches was slightly greater than the main stem (without separation by the means comparison test) and usually similar to the middle main stem (Figures 2F–J). Branch oil content was the same as that of the main stem, with values ranging between the lower and middle segments (Figure 2T). Although protein and oil content did not differ at the whole plant level, segment means (main stem and branches) differed among genotypes, with an evident trade-off for the genotype P34T43R2 (Figures 2L,K). The residue content followed a similar pattern as the protein and oil, with lower values in the lower section of the main stem and greater content in the upper main stem segment (Supplementary Figure 1J). Remarkably, protein was the seed component with the greatest content variation among canopy portions, relative to oil and residue.

### Nutritional Quality of Soybean Seeds (Concentration)

**Figure 3:**
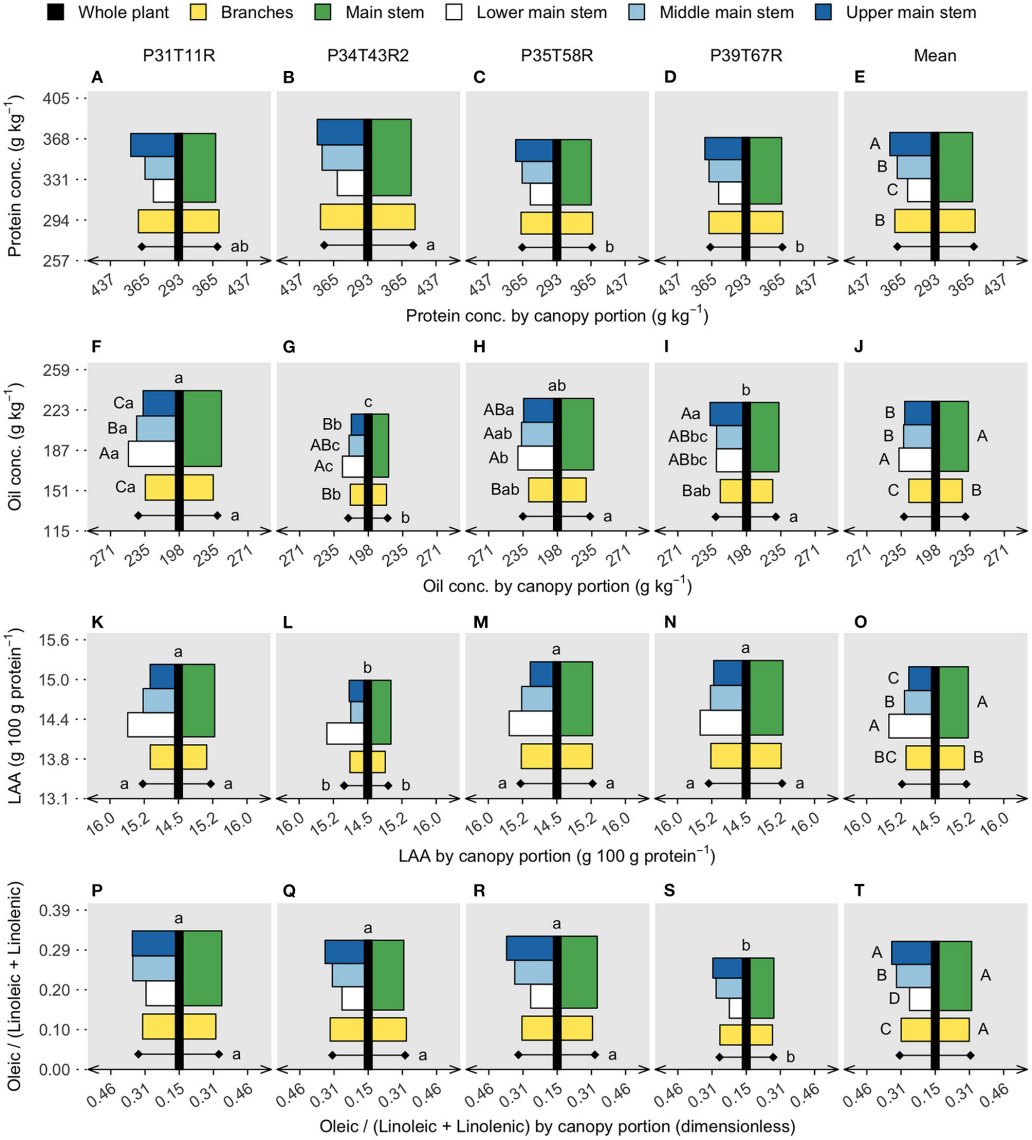
Soybean seed protein **(A–E)** and oil concentration **(F–J)**, limiting amino acids (LAAs) abundance **(K–O)**, and oleic/(linoleic + linolenic) ratio **(P–T)**. Vertical black bars refer to the whole plant data, in the y axis, with lowercase letters comparing genotypes. Horizontal bars are centered on the black bar (x axis), referring to two canopy portions on the right side (main stem and branches) and four canopy portions on the left side (lower, middle, and upper main stem segments and branches). Diamonds represent canopy portion means on each side. Uppercase letters compare canopy portions within genotype (significant interaction) or on the overall mean (canopy portions single effect). Lowercase letters compare genotypes within canopy portions (interaction) or on the genotype mean, diamonds (genotype single effect). The absence of letters represents no significant difference in the analysis of variance (*p* < 0.05).

In the soybean industry, nutritional quality is evaluated in a unit of mass (concentration), not in terms of content per seed. In this scenario, a high concentration can be achieved with increased content of a given component or with decreased content of the other components. For protein, the upper main stem concentration was ~9% greater than the lower main stem, with the middle stem and branches reaching similar values (Figure 3E). Despite genotype interactions, oil concentration was almost 3% greater in the lower main stem than in the upper main stem (Figure 3J). Comparing the entire main stem and branches, protein concentration was similar for those fractions; however, oil decreased from 233 to 227 g/kg, respectively. Genotypes differed on the portion means (main stem and branches) for both protein and oil concentration, with the genotype with the smallest yield (P34T43R2) presenting high protein (Figure 3B) and low oil (Figure 3G) concentrations. Across genotypes, oil concentration ranged from 219 to 240 g/kg and protein concentration ranged from 368 to 386 g/kg. The residue concentration (Supplementary Figure 1O) followed the oil trend, highlighting protein as the seed component with the greatest canopy variation.

Besides protein and oil concentration, soybean nutritional value is determined by protein and oil quality. Here, the protein quality is expressed as the abundance of LAA within a protein and oil as the oleic/(linoleic + linolenic) ratio. Both variables differed across genotypes, with LAA abundance ranging from 15.0 to 15.3 g/100 g protein and the FA ratio ranging from 0.27 to 0.34. The low-yielding genotype (P34T43R2) had the smallest LAA abundance (Figure 3L), while the lower FA ratio was found in the P39T67R (Figure 3S) genotype. Genotypes did not interact with the canopy portion for either LAA or FA. The LAA abundance in the lower stem section was ~3% greater than the upper stem section, while branches were ~0.5% lower than the entire main stem. The oleic/(linoleic + linolenic) ratio was slightly lower in the branches (without separation on the means comparison test), but the upper main stem surpassed the lower segment by ~30%. Because protein decreased from top to bottom main stem segments while LAA increased, the vertical gradient of protein concentration and protein quality was opposite, similar to the oil concentration and quality (measured as the FA ratio).

### Branch-Yield Contribution Affects Seed Composition

The whole plant yield was positively associated with the branch-yield contribution (Figure 1B), increasing about 30 kg/ha when branch contribution increased by 1%. Due to the yield-branch significant relationship, a simple linear regression exploring the effect of branches on seed components would likely confound the two factors. Therefore, protein, oil, LAA concentration, and the oleic/(linoleic + linolenic) ratio were modeled as a function of both variables. Branches had lower oil concentration, LAA abundance, and FA ratio than the main stem (Figure 3). However, when there was more yield coming from branches, the FA ratio was not affected, while oil concentration and LAA abundance increased at the whole plant level (Table 2). For protein, neither yield nor branches had significant slopes. Only yield was found to have a negative relationship with the FA ratio, with more yield meaning poor oil quality. However, for oil concentration and LAA abundance, branch-yield contribution alone was related to greater values, with positive slopes of 0.59 and 0.01, respectively. Considering branch contribution to yield ranged from ~10 to 50%, oil concentration was predicted to increase 23.6 g/kg from low to high branch-yielding conditions. Although the LAA had a smaller rate of change, it represented 0.4 g/100 g protein, matching the overall genotype range presented in Figure 2. The ANOVA coefficients are shown in Supplementary Table 2.

**Table 2:** Whole plant soybean seed protein, oil, and limiting amino acids (LAAs) concentration, and oleic/(linoleic + linolenic) ratio as a function of whole plant yield (Mg/ha) and branch contribution to the whole plant yield (%).

**Parameter**	**Estimate**	**Standard error**	***p*-value**	**Back transformed slope estimate**
Protein, g kg^−1^				
Intercept	373.6	11.65	2.21e-02*	
Whole plant yield	−0.02	3.53	9.96e-01	−0.02
Branch contribution	−5.74	3.39	1.06e-01	−0.51
Oil, g kg^−1^				
Intercept	231.7	5.17	1.95e-02*	
Whole plant yield	−3.79	2.58	1.56e-01	−5.10
Branch contribution	6.63	2.49	1.48e-02*	0.59
LAA, g 100 g protein^−1^				
Intercept	15.21	0.35	1.50e-02*	
Whole plant yield	0.02	0.05	6.98e-01	0.02
Branch contribution	0.10	0.04	3.77e-02*	0.01
Oleic/(Linoleic + Linolenic)				
Intercept	0.31	0.02	5.62e-02	
Whole plant yield	−0.02	0.01	2.55e-02*	−0.03
Branch contribution	0.01	0.01	4.23e-01	0.00

Observed variables were centered and scaled in the linear model; however, slopes were finally back transformed to the original variable units. *Estimate is significant at the 0.05 probability level (p < 0.05).

## Discussion

Results from this study showed the importance of branch-yield contribution for soybean seed composition, expanding previous findings on protein and oil vertical gradient in the main stem. Additionally, this study provides novel analysis, including a characterization of the abundance of limiting AA and oleic/(linoleic + linolenic) ratio, as parameters of protein and oil quality. We acknowledge some limitations of this study, such as the limited number of tested genotypes and collinearity between yield and branch-yield contribution. However, manipulating branches under field conditions while attaining comparable seed yield is difficult and highly sensitive to genotype, environment, and management (G × E × M) interactions.

A vertical gradient of seed protein and oil concentration was expected. Escalante and Wilcox (1993a) found more protein in the upper main stem (~40 g/kg) than in the lower main stem. Additionally, the same authors documented genotypes with contrasting protein concentration increased ~7 g/kg/node from lower to upper main stem (Escalante and Wilcox, 1993b). Bellaloui and Gillen (2010) found more protein and less oil concentrations in the upper nodes, with differences attributed to changes in genotype and light distribution within the canopy. Sharma et al. (2013) observed the same protein-oil vertical gradient going beyond physiological maturity and affecting composition during storage. Our results confirm the main stem vertical gradient widely reported, with protein concentration decreasing (~9%) and oil concentration increasing (~3%) both from top to bottom main stem nodes. However, greater oil concentration in the lower nodes was not associated with greater seed oil content but with a proportionally greater reduction in protein content than both oil and residue compounds. Protein was the seed component with the greatest variation among canopy portions, pointing to protein accumulation as a critical process determining the concentration of others seed components within the plant.

Assimilate supply and ontogenesis temporal variation might be the complementary factors defining protein and oil content among nodes. Our genotypes presented slightly greater protein content in the branches than the main stem and greater seed size in the upper nodes than the lower nodes. Indeterminate soybean genotypes start setting pods from lower to upper nodes (Egli and Bruening, 2006a) and later in branches than the main stem (Munier-Jolain et al., 1994). However, a possibly shorter seed-filling period might be compensated by a greater accumulation rate (Egli et al., 1978), since seeds from upper nodes are not necessarily smaller (Parvej et al., 2016), and there is no clear association between timing of fruit initiation and seed size (Egli, 2012). Greater seed size might be connected to CO_2_ assimilation and N concentration in upper leaves (Boon et al., 1983), as the protein content in the branches could be driven by greater light exposure relative to the lower main stem. On the other hand, considering protein is accumulated before oil (Poeta et al., 2014), a shorter seed-filling duration in branches and upper nodes could limit the oil deposition, affecting its overall final accumulation (evidenced by a relative stability of oil compared to protein).

Greater protein quality (LAA abundance) was found in the lower main stem, while greater oil quality (FA ratio) was found in the upper main stem. These differences might be related to microclimatic canopy changes, especially temperature and light (both are greater in the upper canopy). Higher temperatures promote the synthesis of oleic acid at the cost of linoleic and linolenic (Wolf et al., 1982). Because LAAs are less dependent on carbon supply, their abundance is maintained or increased under shading conditions (Pfarr et al., 2018). Within the plant, low mobility of sulfur could enhance the differences on LAA along the main stem (Sexton et al., 2002), since two out of the five LAAs are rich in sulfur (cysteine and methionine). Regarding branches, our results confirm the expected FA trend, with a lower oleic/(linoleic + linolenic) ratio, possibly due to reduced temperature compared to the upper main stem. However, branches presented a smaller LAA abundance relative to the entire main stem, possibly indicating greater light exposure than the lower main stem nodes, decreasing the abundance of LAA (Pfarr et al., 2018).

Our results point to branch yield as an underlying factor of seed composition. It is possible that a greater branch yield favors the accumulation of oil and LAA at the whole plant level, even though branch seeds have a lower concentration of those components. The importance of branch yield for seed yield formation and stability has been highlighted by Carpenter and Board (1997) and Suhre et al. (2014), but information on seed quality changes was lacking. From our study, we demonstrated that more branch yield drove the whole plant seed composition toward the lower main stem characteristics (high oil concentration and LAA abundance); however, the overall branch seed composition is not similar to the lower main stem. Changes in plant density and arrangement could affect the branch length and microclimate within the canopy, making branch seeds similar to either lower or middle-upper main stem segments. However, under a reduced row spacing (0.45 m) branch seed composition was consistent with our results (Werner et al., 2021).

Changing the yield allocation among canopy portions could be manipulated by plant breeding and management practices. For instance, enhanced branching in modern genotypes could be related to stable LAA concentration despite protein decay over the last 40 years (de Borja Reis et al., 2020). Future research must consider to study the branching process (initiation, progress rate and duration, dry matter, and harvest index) as a potential moderator of soybean seed composition, plausibly benefitting human nutrition with improved AA profile and oil concentration. Findings of this study denote two relevant points from a breeding standpoint: (i) expanding assessment on seed quality to include not only protein and oil but also AA and FA and (ii) acknowledging the contribution of the breeding process on branching, exploiting genetic variation on this trait to assess potential improvements in seed quality.

## Conclusions

In conclusion, soybean seeds from the upper main stem segment accumulated more protein and oil than the lower main stem segment. However, the upper main stem section presented greater protein and lower oil concentrations relative to the lower main stem section of this segment. Across genotypes with contrasting branch yield, seeds from the branches presented similar protein concentration as the main stem but lower oil concentration, oleic/(linoleic + linolenic) ratio, and LAA abundance. However, branch-yield contribution was related to greater oil concentration and LAA abundance across genotypes. This study highlights the importance of improving our knowledge on yield contribution and seed composition from different canopy portions for benefitting food production and soybean markets.

## Data Availability Statement

The raw data supporting the conclusions of this article will be made available by the authors, without undue reservation.

## Author Contributions

LM: conceptualization, methodology, formal analysis, investigation, writing original draft, visualization, and project administration. AB: conceptualization, methodology, formal analysis, and writing original draft. IC: conceptualization, methodology, formal analysis, resources, writing original draft, supervision, project administration, and funding acquisition. All authors contributed to the article and approved the submitted version.

## Conflict of Interest

The authors declare that the research was conducted in the absence of any commercial or financial relationships that could be construed as a potential conflict of interest.

## Publisher's Note

All claims expressed in this article are solely those of the authors and do not necessarily represent those of their affiliated organizations, or those of the publisher, the editors and the reviewers. Any product that may be evaluated in this article, or claim that may be made by its manufacturer, is not guaranteed or endorsed by the publisher.
