# Supplementary material for: Vertical Canopy Profile and the Impact of Branches on Soybean Seed Composition
Source: Front Plant Sci. 2021 Sep 8;12:725767. doi: 10.3389/fpls.2021.725767 (PMC8457254; doi:10.3389/fpls.2021.725767)
Supplement: Supplementary file 1 [file Data_Sheet_1.PDF]

Supplementary Material

1 Supplementary Tables

Table S1. Analysis of variance (ANOVA) for soybean traits at harvest time. Whole plant analysis tests the effect of genotype. Two portion analyses also compared main stem and branches. Four canopy portions include lower, middle, upper main stem, and branches. The number within parentheses represents degrees of freedom, followed by the p-value (F-test).

	**Whole plant**	**Two canopy portions**			**Four canopy portions**		
**Variable**	**Genotype**	**Segment**	**Genotype**	**Interaction**	**Segment**	**Genotype**	**Interaction**
Yield, Mg ha^{-1}	(3) 3.29e-02 *	(1) 2.67e-06 ***	(3) 7.77e-04 ***	(3) 1.14e-02 *	(3) 4.84e-02 *	(3) 3.43e-08 ***	(9) 1.49e-04 ***
Size, mg seed^{-1}	(3) 8.02e-01	(1) 5.84e-02	(3) 7.87e-02	(3) 4.35e-02 *	(3) 1.53e-08 ***	(3) 2.62e-01	(9) 2.82e-02 *
Number, seeds m^{-2}	(3) 1.89e-02 *	(1) 5.20e-07 ***	(3) 6.46e-04 ***	(3) 1.02e-02 *	(3) 4.74e-01	(3) 4.79e-09 ***	(9) 2.80e-05 ***
Ol / (Li + Ln) [a]	(3) 4.21e-03 **	(1) 1.29e-02 *	(3) 2.26e-02 *	(3) 9.35e-02	(3) 1.10e-03 **	(3) 9.04e-02	(9) 5.43e-01
Content, mg seed^{-1}							
Protein	(3) 3.78e-01	(1) 2.10e-02 *	(3) 1.49e-02 *	(3) 4.10e-02 *	(3) 1.79e-10 ***	(3) 9.40e-02	(9) 2.97e-02 *
LAA [b]	(3) 4.97e-01	(1) 4.83e-02 *	(3) 2.84e-02 *	(3) 7.30e-02	(3) 2.69e-10 ***	(3) 1.47e-01	(9) 2.17e-02 *
EAA [c]	(3) 3.82e-01	(1) 2.18e-02 *	(3) 1.41e-02 *	(3) 3.51e-02 *	(3) 2.69e-10 ***	(3) 9.42e-02	(9) 2.42e-02 *
NAA [d]	(3) 3.96e-01	(1) 2.90e-02 *	(3) 1.71e-02 *	(3) 4.93e-02 *	(3) 2.42e-10 ***	(3) 1.08e-01	(9) 3.46e-02 *
Oil	(3) 1.29e-01	(1) 8.98e-01	(3) 4.30e-02 *	(3) 6.82e-02	(3) 4.54e-06 ***	(3) 1.08e-01	(9) 6.37e-02
Oleic	(3) 7.90e-03 **	(1) 1.64e-01	(3) 1.26e-02 *	(3) 4.81e-02 *	(3) 2.12e-05 ***	(3) 3.53e-02 *	(9) 6.59e-01
Linoleic	(3) 8.27e-02	(1) 1.53e-01	(3) 3.10e-02 *	(3) 3.14e-02 *	(3) 1.27e-05 ***	(3) 7.55e-02	(9) 1.19e-03 **
Linolenic	(3) 5.10e-01	(1) 7.96e-01	(3) 1.68e-01	(3) 4.94e-01	(3) 3.81e-01	(3) 2.21e-01	(9) 6.35e-01
Residual	(3) 9.39e-01	(1) 4.42e-02 *	(3) 6.39e-01	(3) 1.23e-01	(3) 4.45e-06 ***	(3) 8.11e-01	(9) 2.81e-02 *
Concentration, g kg^{-1}							
Protein	(3) 1.16e-01	(1) 6.49e-02	(3) 2.00e-02 *	(3) 6.79e-01	(3) 5.39e-11 ***	(3) 6.45e-02	(9) 1.30e-01
LAA	(3) 2.03e-01	(1) 4.07e-01	(3) 7.02e-02	(3) 9.33e-01	(3) 9.76e-11 ***	(3) 1.68e-01	(9) 1.27e-01
EAA	(3) 8.77e-02	(1) 1.14e-01	(3) 1.32e-02 *	(3) 7.26e-01	(3) 4.17e-10 ***	(3) 5.79e-02	(9) 1.81e-01
NAA	(3) 1.40e-01	(1) 1.52e-01	(3) 2.67e-02 *	(3) 8.06e-01	(3) 1.45e-10 ***	(3) 8.56e-02	(9) 1.78e-01
Oil	(3) 5.26e-03 **	(1) 9.80e-03 **	(3) 3.69e-02 *	(3) 5.63e-01	(3) 6.82e-06 ***	(3) 6.22e-02	(9) 1.22e-02 *
Oleic	(3) 4.15e-03 **	(1) 9.64e-03 **	(3) 5.47e-02	(3) 1.21e-01	(3) 1.95e-02 *	(3) 1.20e-01	(9) 1.99e-01
Linoleic	(3) 7.01e-04 ***	(1) 3.41e-01	(3) 5.22e-04 ***	(3) 8.03e-01	(3) 3.20e-06 ***	(3) 1.06e-03 **	(9) 1.09e-01
Linolenic	(3) 3.26e-01	(1) 4.41e-01	(3) 3.97e-01	(3) 7.72e-01	(3) 1.16e-02 *	(3) 4.13e-01	(9) 7.41e-01
Residual	(3) 3.53e-02 *	(1) 5.68e-01	(3) 5.69e-03 **	(3) 7.42e-02	(3) 2.68e-08 ***	(3) 2.57e-02 *	(9) 1.37e-01
Concentration, g 100 g protein^{-1}							
LAA	(3) 3.25e-02 *	(1) 7.14e-04 ***	(3) 5.22e-03 **	(3) 6.32e-02	(3) 2.83e-08 ***	(3) 1.49e-02 *	(9) 1.30e-01
EAA	(3) 6.15e-02	(1) 9.47e-02	(3) 7.81e-02	(3) 7.25e-01	(3) 8.68e-03 **	(3) 1.67e-01	(9) 9.72e-01
NAA	(3) 4.88e-01	(1) 9.16e-02	(3) 1.65e-01	(3) 5.25e-01	(3) 2.06e-01	(3) 2.72e-01	(9) 5.91e-01
Concentration, g 100 g oil^{-1}							
Oleic	(3) 4.84e-03 **	(1) 4.33e-02 *	(3) 2.22e-02 *	(3) 1.39e-01	(3) 2.46e-03 **	(3) 7.18e-02	(9) 5.41e-01
Linoleic	(3) 5.55e-05 ***	(1) 2.26e-03 **	(3) 1.09e-04 ***	(3) 2.56e-02 *	(3) 1.74e-03 **	(3) 1.43e-03 **	(9) 4.80e-02 *
Linolenic	(3) 7.36e-03 **	(1) 8.65e-01	(3) 4.86e-02 *	(3) 8.28e-01	(3) 1.40e-01	(3) 4.26e-02 *	(9) 9.59e-01

[a] Ratio between oleic / (linoleic + linolenic) fatty acids. [b] Five limiting (LAA) amino acids (lysine, cysteine, methionine, threonine, and tryptophan). [c] Five essential non limiting (EAA) amino acids (isoleucine, leucine, histidine, phenylalanine, and valine). [d] Ten non-essential (NAA) amino acids: alanine, arginine, aspartic acid (asparagine and aspartate), glutamic acid (glutamine and glutamate), glycine, proline, serine, and tyrosine. * p-value < 0.05. ** p-value < 0.01. *** p-value < 0.001.

Table S2. Analysis of variance (ANOVA) for the relationship between seed protein, oil, limiting amino acids (LAA) abundance, oleic / (linoleic + linolenic) ratio and the whole plant seed yield (Mg ha^{-1}) and branch yield contribution to the whole plant (%). Both observed continuous variables were centered and scaled in the model. The whole plant model corresponds to the whole plant values of the response variables. A model comparing the four canopy portions (lower, middle, upper main stem, and branches) confirms if the whole plant trends are stable within the plant (absence of interaction). The number within parentheses represents degrees of freedom (df), followed by the p-value (F-test).

**Factor**	**Protein (g kg^{-1})**	**Oil (g kg^{-1})**	**LAA (g 100 g $protein^{-1}$)**	**Ol / (Li + Ln)**
Whole plant				
Intercept	(1) 2.21e-02 *	(1) 1.95e-02 *	(1) 1.50e-02 *	(1) 5.62e-02
Whole plant yield (WY)	(1) 9.97e-01	(1) 1.82e-01	(1) 7.04e-01	(1) 3.35e-02 *
Branch contribution (BC)	(1) 1.17e-01	(1) 1.98e-02 *	(1) 4.45e-02 *	(1) 4.36e-01
Four stem segments				
Intercept	(1) 1.22e-02 *	(1) 5.69e-03 **	(1) 1.24e-02 *	(1) 2.95e-02 *
Stem segment	(3) 6.33e-13 ***	(3) 4.06e-03 **	(3) 4.17e-12 ***	(3) 6.35e-08 ***
Whole plant yield (WY)	(1) 7.59e-01	(1) 1.03e-01	(1) 2.25e-01	(1) 4.82e-02 *
Branch contribution (BC)	(1) 1.13e-01	(1) 3.98e-02 *	(1) 1.37e-02 *	(1) 8.93e-01
Segment × WY	(3) 3.38e-01	(3) 2.73e-01	(3) 1.32e-01	(3) 6.16e-01
Segment × BC	(3) 4.47e-01	(3) 6.04e-01	(3) 5.63e-01	(3) 7.43e-01

* Significant at the 0.05 probability level. ** Significant at the 0.01 probability level. *** Significant at the 0.001 probability level.

1.1 Supplementary Figures

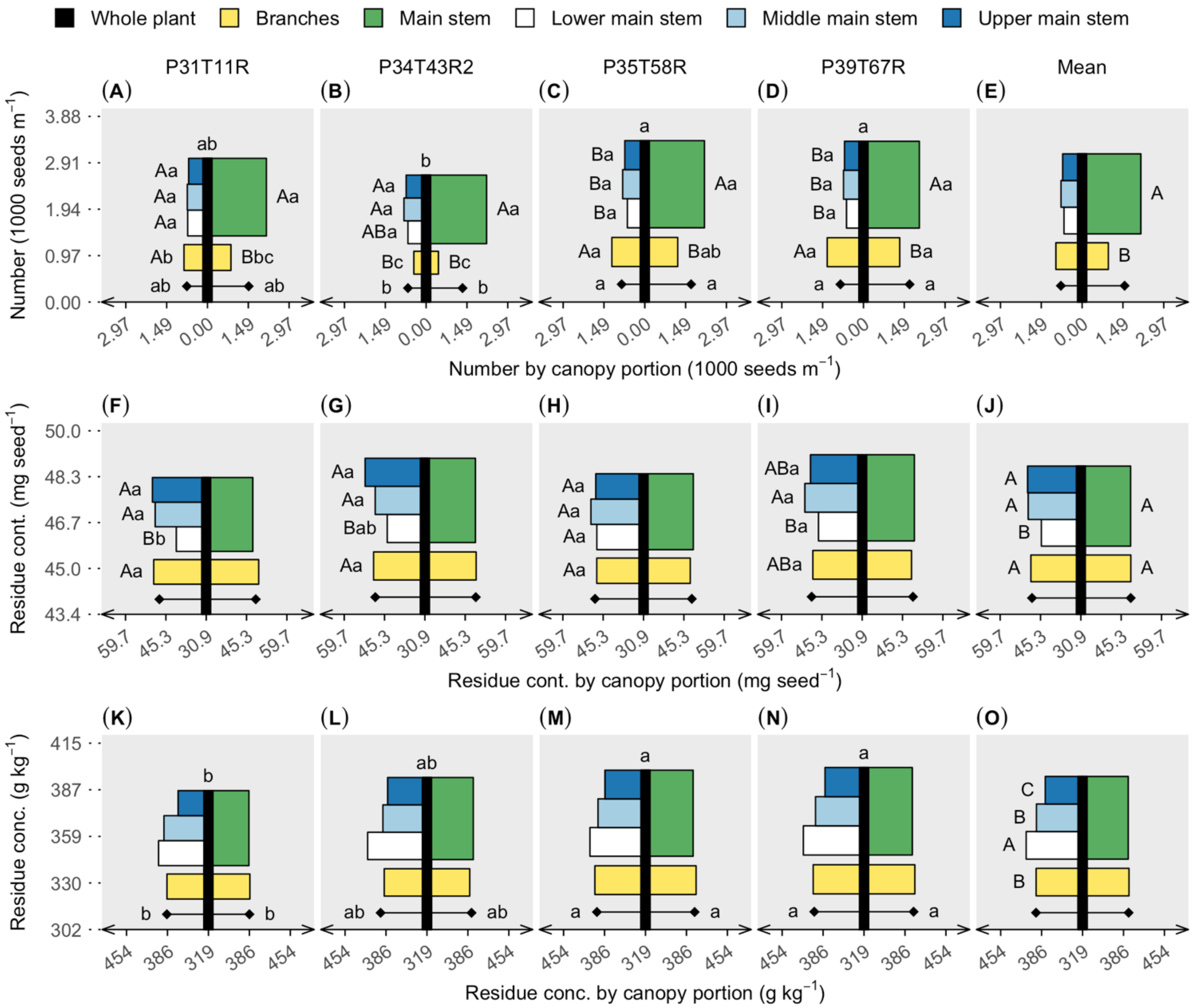

Figure S1. Soybean seed number, residue content and concentration by genotype and overall mean. Residue is the remaining seed size after accounting for protein and oil. Vertical black bars refer to the whole plant data in the y axis, with lowercase letters comparing genotypes. Horizontal bars are centered on the black bar (x axis), referring to two canopy portions on the right side (main stem and branches) and four on the left side (lower, middle, upper main stem, and branches). Diamonds represent the genotype mean of canopy portions on each side. Uppercase letters compare portions within genotype (significant interaction) or on the overall mean (portion effect). Lowercase letters compare genotypes within canopy portions (interaction) or on the genotype mean, diamonds (genotype effect). Each row of panels portraits three linear models of a response variable, for the whole plant, two and four stem segments. Absence of letters represent $p < 0.05$.
